# “Metalloestrogenic” effects of cadmium downstream of G protein-coupled estrogen receptor and mitogen-activated protein kinase pathways in human uterine fibroid cells

**DOI:** 10.1007/s00204-021-03033-z

**Published:** 2021-04-05

**Authors:** Linda Yu, Jingli Liu, Yitang Yan, Alanna Burwell, Lysandra Castro, Min Shi, Darlene Dixon

**Affiliations:** 1 Molecular Pathogenesis Group, Mechanistic Toxicology Branch, Division of the National Toxicology Program (DNTP), National Institute of Environmental Health Sciences (NIEHS), National Institutes of Health (NIH), MDB3-06/B341, 111 T.W. Alexander Drive, Bldg.101, South Campus, P.O. Box 12233, Research Triangle Park, NC 27709 USA; 2 Biostatistics and Computational Biology Branch, Division of the Intramural Program (DIR), National Institute of Environmental Health Sciences (NIEHS), National Institutes of Health (NIH), Research Triangle Park, NC 27709 USA

**Keywords:** Aurora B, H3Ser10ph, FOXM1, Cyclin D1, Fibroid cells

## Abstract

Cadmium (Cd) is a toxic metal reported to act as an estrogen “mimic” in the rat uterus and in vitro. We have reported that Cd stimulates proliferation of estrogen-responsive human uterine leiomyoma (ht-UtLM; fibroid) cells through nongenomic signaling involving the G protein-coupled estrogen receptor (GPER), with activation of epidermal growth factor receptor (EGFR) and mitogen-activated protein kinase (pMAPK44/42). In this study, we explored Cd-induced mechanisms downstream of MAPK and whether Cd could stimulate phosphorylation of Histone H3 at serine 10 (H3Ser10ph) through activated Aurora B kinase (pAurora B), a kinase important in activation of histone H3 at serine 10 during mitosis, and if this occurs via Fork head box M1 (FOXM1) and cyclin D1 immediately downstream of MAPK. We found that Cd increased proliferating cell nuclear antigen (PCNA) and H3Ser10ph expression by immunofluorescence, and that H3ser10ph and pAurora B were coexpressed along the metaphase plate in ht-UtLM cells. In addition, Cd-exposed cells showed higher expression of pMAPK44/42, FOXM1, pAurora B, H3ser10ph, and Cyclin D1 by western blotting. Immunoprecipitation and proximity ligation assays further indicated an association between FOXM1 and Cyclin D1 in Cd-exposed cells. These effects were attenuated by MAPK kinase (MEK1/2) inhibitor. In summary, Cd-induced proliferation of ht-UtLM cells occurred through activation of Histone H3 and Aurora B via FOXM1/Cyclin D1 interactions downstream of MAPK. This provides a molecular mechanism of how Cd acts as an “estrogen mimic” resulting in mitosis in hormonally responsive cells.

## Introduction

Cd is an element from the earth’s crust that is toxic and has high rates of soil-to-plant transference (Satarug 2018). Cd exposure can occur through food consumption, cigarette smoke, smelting, or mining workplaces that generate Cd (Faroon et al. 2012). Recently, Cd has been proposed to act as an environmental endocrine disruptor (Gao et al. 2015; Johnson et al. 2003; Kluxen et al. 2012), or an estrogen “mimic” in the rat uterus (Johnson et al. 2003), and Cd blood concentrations correlate with estrogen receptor levels in women with uterine fibroids (Nasiadek et al. 2011; Ye et al. 2017). We have previously reported that Cd induces increased cell proliferation of human uterine leiomyoma (fibroid) cells through nongenomic activation of the ERK/MAPK pathway (Gao et al. 2015), and that Cd does not significantly bind to estrogen receptor alpha or beta, nor does it show transactivation in the cells transiently transfected with Estrogen Responsive Element (ERE) luciferase reporters; however, we found Cd-induced activation of GPER1 with mediation of matrix metalloproteinase 1&2 (MMP1&2) and EGFR in vitro (Gao et al. 2015; Liu et al. 2019). Uterine fibroids are common hormonally responsive tumors that clinically affect 25–30% of premenopausal US women, occur in over 70% of  US women by menopause, and are reported in women worldwide (Cramer and Patel 1990; Segars et al. 2014). Cd has been found in fibroid tumors in women (Nasiadek et al. 2011) and in women of reproductive age, there is a statistically significant association between blood Cd levels and fibroid volume (Ye et al. 2017). In addition, we have found that Cd induces proliferation of human fibroid cells through MAPK activation in vitro and were interested in determining the effects of Cd on the molecular signaling events downstream of MAPK activation.

The phosphorylation of Histone H3 at serine 10 is a post-translational modification event important in mitosis (Prigent and Dimitrov 2003) and Aurora B kinase is a kinase found to play an important role in the phosphorylation of histone H3 at serine 10 during mitosis (Emanuele et al. 2008; Hirota et al. 2005). There are also studies that show the cyclin D1 and FOXM1 axis unequivocally plays a pivotal role in the regulation of cellular proliferation and transformation through phosphorylation of Aurora B (Bonet et al. 2012; Liao et al. 2018; Manzione et al. 2020) during the G2/M phase of the cell cycle, and that FOXM1 and Aurora B are coexpressed in tumors (Huang et al. 2015). Therefore, this study was designed to further evaluate the downstream molecular signaling events mediated by MAPK through EGFR activation in Cd-exposed cells (Gao et al. 2015; Liu et al. 2019); and to determine whether the proliferation of ht-UtLM cells induced by Cd could occur through phosphorylation of Histone H3 by Aurora B phosphorylation via FOXM1/Cyclin D1 activation downstream of MAPK. These findings will delineate a downstream molecular mechanism of Cd-induced cell proliferation in hormonally responsive human uterine fibroid cells.

## Results

### Cd exposure of 24 h increased H3Ser10ph expression in ht-UtLM cells

**Fig. 1:**
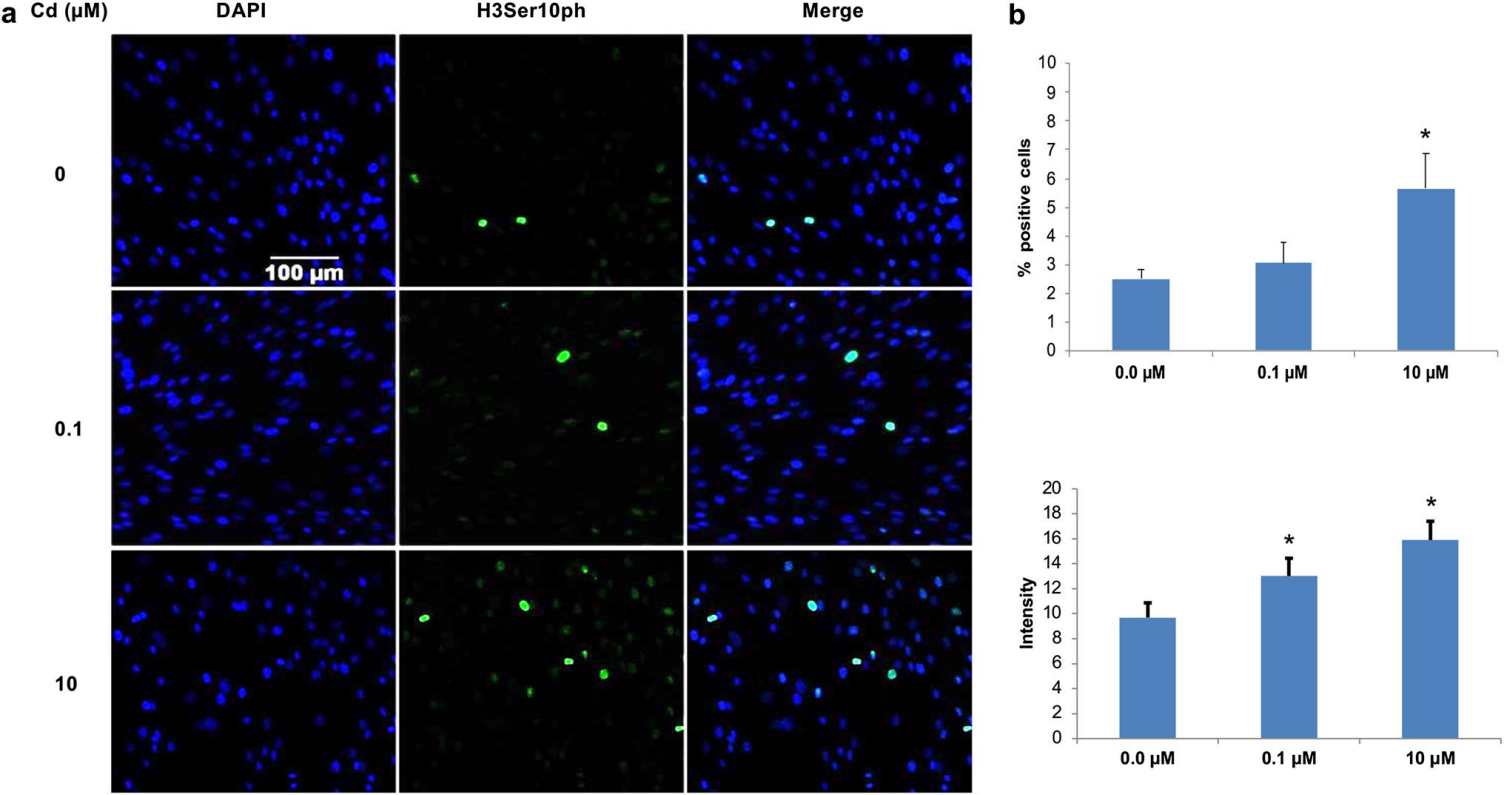
Confocal immunofluorescence staining of phosphorylated Histone H3 at serine 10 (H3Ser10ph) in Cd-exposed cells. Ht-UtLM cells exposed to Cd for 24 h at 0.1 µM and 10 µM induced H3Ser10ph expression. **a** Fluorescence staining of H3Ser10ph. Both Cd-treated or nontreated ht-UtLM cells expressed H3Ser10ph, which was located in the cell nucleus (green). **b** The intensity and percentage of positive cells. H3Ser10ph positively stained cells increased at 0.1 and 10 µM of Cd compared to control cells (0.0 μM, *P < 0.04), the intensity of the staining also significantly (*P < 0.01) increased at 0.1 and 10 µM. Data represent the mean ± SEM of percentage (%) of positive staining area and intensity from scanned images. Scale bar for all images = 100 µm

Since phosphorylation of histone H3 at serine 10 site is an important modification involved in DNA decondensation, transcription, and cell division (Prigent and Dimitrov 2003), immunofluorescence staining was used to measure the expression level of H3Ser10ph when ht-UtLM cells were exposed to Cd (Fig. 1a). H3Ser10ph expression was localized to the nuclei of uterine fibroid cells. There was an increased percentage of H3Ser10ph-positive cells following Cd treatment at 0.1 (3.05%) or 10 µM (5.670%, *p* < 0.04) at 24 h compared to the control cells (0 µM, 2.49%) (Fig. 1b). The intensity of the fluorescence signal of H3Ser10ph was also significantly increased (*p* < 0.01) in the cells exposed to Cd at 0.1 (13.03) or 10 µM (15.90) for 24 h versus the control cells (0 µM, 9.70) (Fig. 1b).

### H3Ser10ph and Aurora B colocalized in ht-UtLM cells exposed to Cd for 24 h

**Fig. 2:**
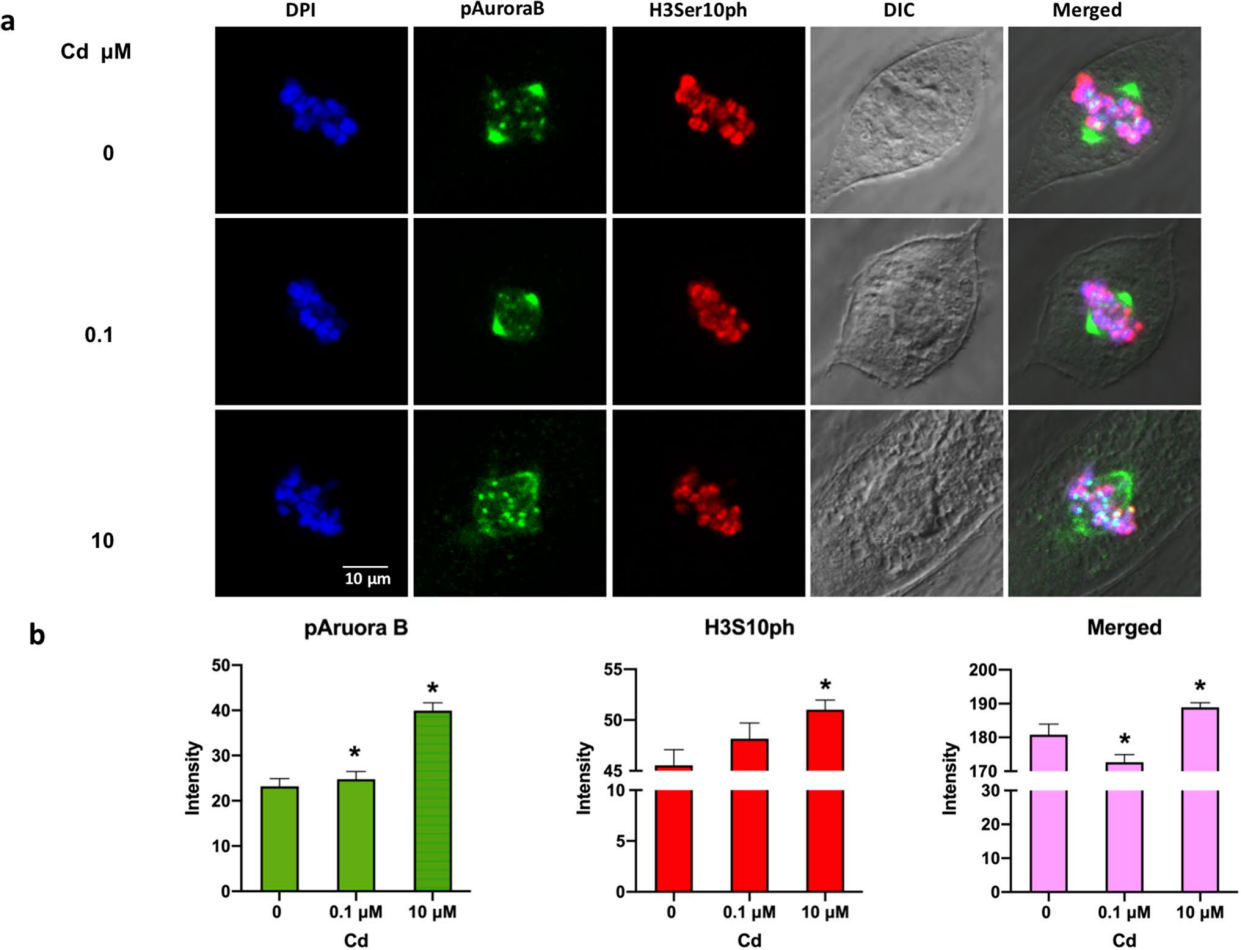
Colocalization of phosphorylated Histone H3 at serine 10 (H3Ser10ph) and Aurora Kinase B (pAurora B) induced by Cd in ht-UtLM cells. Ht-UtLM cells exposed to Cd for 24 h at 0.1 µM and 10 µM induced H3Ser10ph (red) and pAurora B (green, at metaphase plate) expression and colocalization (yellow) in ht-UtLM cells (**a**). Both proteins were highly phosphorylated (*P < 0.05) and merged at the metaphase plate during mitosis (*P < 0.02) of the cells (**b**). Differential Interference Contrast (DIC). Scale bar for all images = 10 µm

Due to the increased expression levels of H3Ser10ph observed in the Cd-exposed ht-UtLM cells, an event that might be activated by Aurora B, we determined the association of H3Ser10ph with Aurora B through colocalization of phosphorylated Aurora B and H3Ser10ph using immunofluorescence staining. The confocal microscopy revealed Aurora B and H3Ser10ph were predominantly localized to the nuclei of the ht-UtLM cells and both proteins were highly phosphorylated and coexpressed at the metaphase plate (Fig. 2a) during mitosis when cells were treated with Cd for 24 h. The intensity of the pAurora B staining was increased at 0.1 (24.76, *p* < 0.05) or 10 µM (39.93, *p* < 0.01) compared to the control cells (0 µM, 23.17) (Fig. 2b). The intensity of the H3Ser10ph staining was also increased at 0.1 (48.15) or 10 µM (51.03, *p* < 0.02) compared to the control cells (0 µM, 45.53) (Fig. 2b). The merged intensity of pAurora B and H3Ser10ph was 172.66 at 0.1 µM (*p* < 0.02) and 188.93 at 10 µM (*p* < 0.02) compared to the control cells (0 µM, 180.83) (Fig. 2b).

### MAPK inhibition by PD decreased Cd-induced PCNA expression

**Fig. 3:**
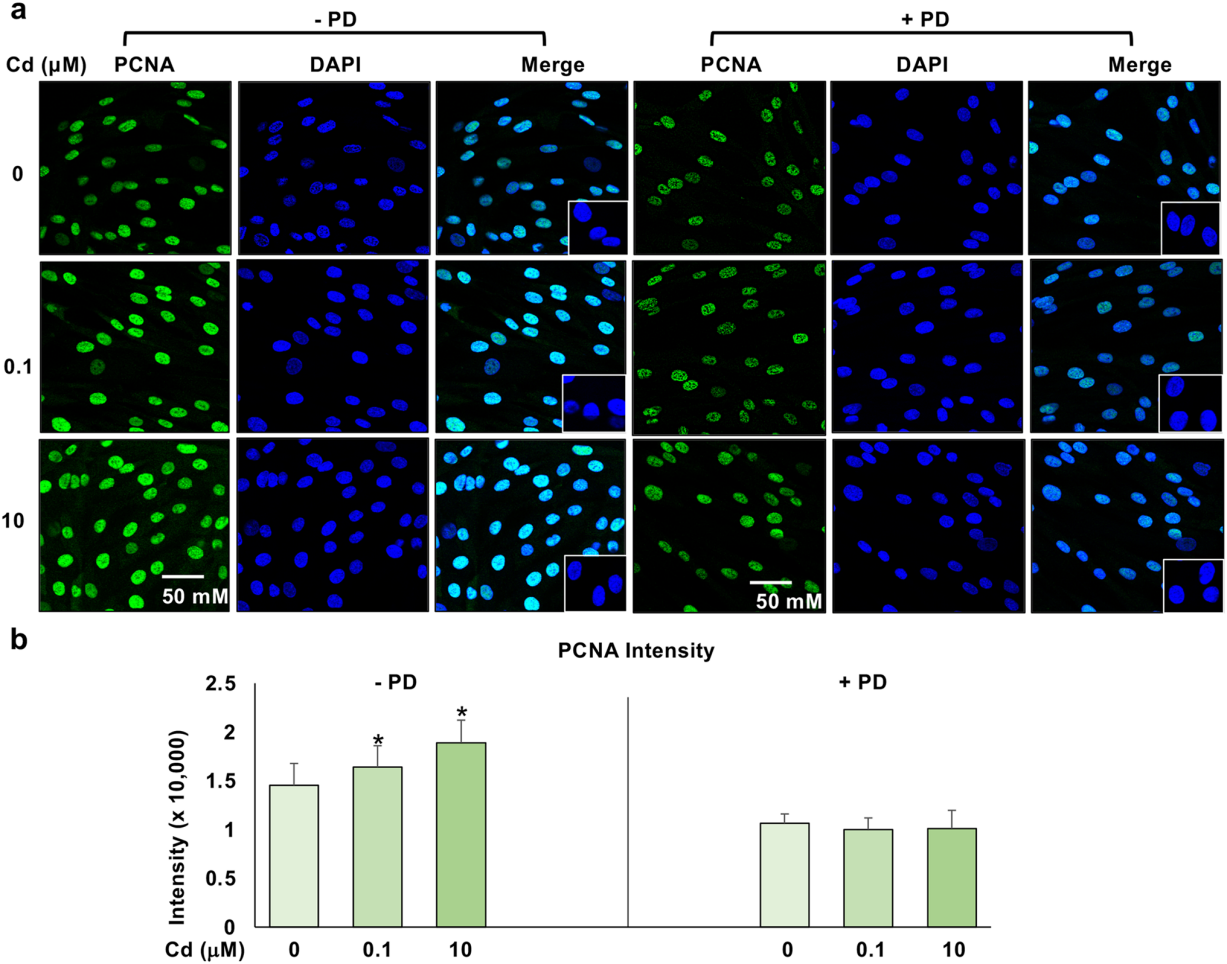
Assessment of cell proliferation based on proliferating cell nuclear antigen (PCNA) expression induced by Cd. Ht-UtLM cells exposed to Cd for 24 h at 0.1 µM and 10 µM induced PCNA expression that was localized in the nuclei of cells. **a** PCNA expression in ht-UtLM cells. Immunofluorescence expression of PCNA was seen in both Cd-exposed and non-exposed ht-UtLM cells and located in the nucleus of the cells (PCNA = green, nucleus = blue). **b** PCNA staining intensity. The intensity of PCNA was significantly (*P* < 0.05) higher in Cd-exposed ht-UtLM cells (0.1 or 10 µM) compared to the cells without Cd exposure (0 µM). The MAPK/MEK1/2 inhibitor, PD attenuated the effects. Data represent the mean ± SEM of staining intensity from 6 scanned areas. Scale bar for all images = 50 µm. Inset = Negative control, rabbit or mouse IgG

In a previous study, we found that Cd could increase ht-UtLM cell proliferation using MTS assays at a concentration range of 0.1–20 μM with concurrent increased expression of MAPK (Gao et al. 2015). In this study, we confirmed that the proliferative effects of Cd through the measurement of proliferating cell nuclear antigen (PCNA), a marker of cell replication, was dependent on MAPK activation. The ht-UtLM cells were cultured with Cd at 0.1 and 10 μM for 24 h with or without the MAPK/MEK1/2 inhibitor, PD, and stained with PCNA antibody. The expression of PCNA was present in both Cd-exposed and non-exposed ht-UtLM cells (Fig. 3a). However, there was significantly increased intensity of PCNA staining (Fig. 3a) in the nuclei of ht-UtLM cells exposed to Cd at 0.1 (1.64 × 10^4^) or 10 µM (1.89 × 10^4^) of Cd for 24 h versus control cells (0 µM, 1.45 × 10^4^) (*p* < 0.05, Fig. 3b). PD treatment attenuated the increased level of PCNA expression and intensity of PCNA nuclear staining (Fig. 3a) induced by Cd at 0.1 µM (0.99 × 10^4^) and 10 µM (1.01 × 10^4^) at 24 h. The results confirmed the effect of Cd on regulation of markers of cell proliferation is dependent on the presence of MAPK and supports our previous findings (Gao et al. 2015).

### Cd exposure (0.1 µM and 10 µM) for 10 min increased activated protein expression

**Fig. 4:**
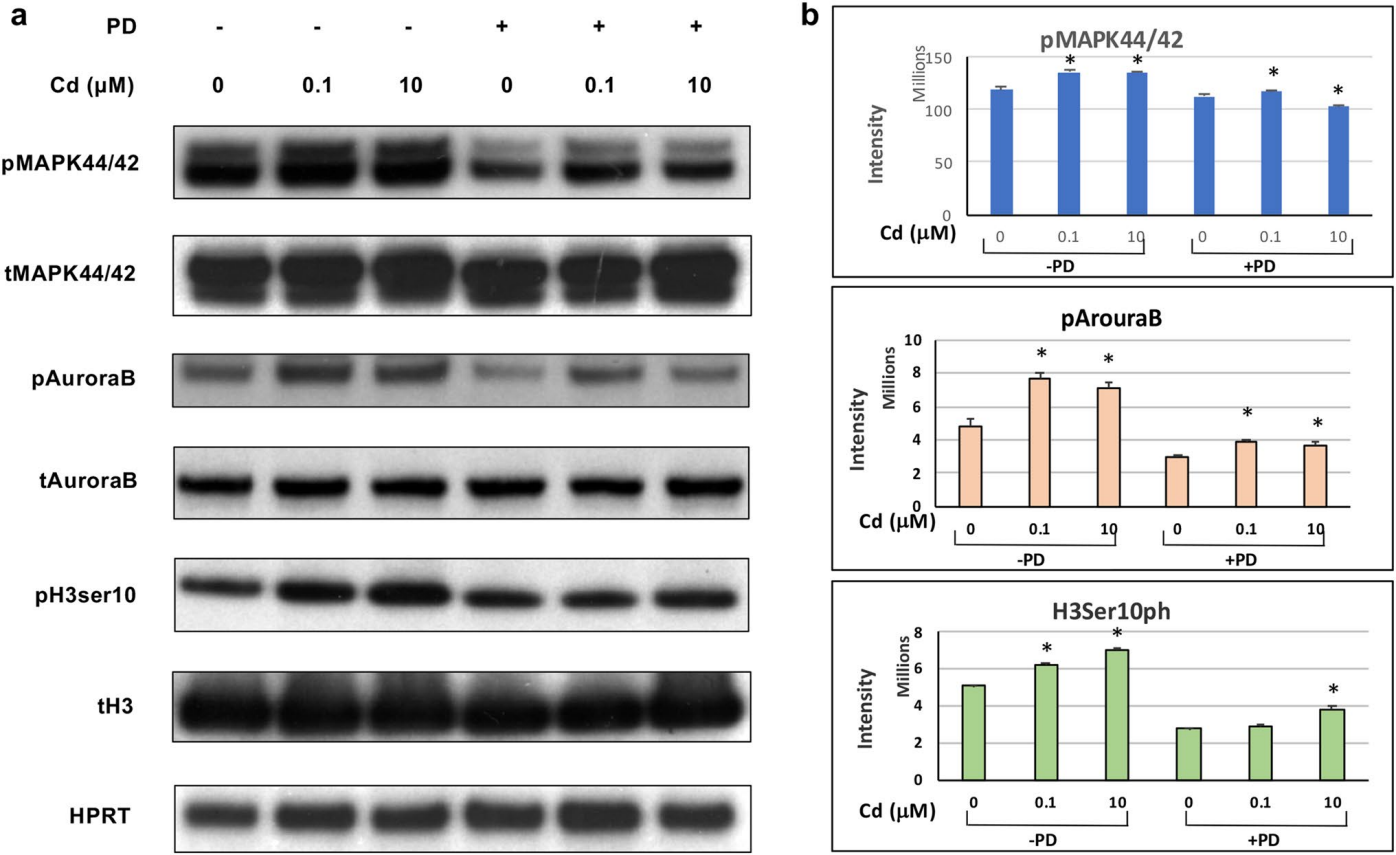
Western blots of pMAPK44/42, pAurora B and H3Ser10ph in Cd-treated ht-UtLM with or without MAPK/MEK1/2 inhibitor, PD. **a** Images of western blotting. **b** Graphs of band intensities. There was a significant increase in the expression of pMAPK44/42, pAurora B and H3Ser10ph in Cd-treated ht-UtLM cells (*P < 0.01) compared to untreated cells. There were no differences observed in the expression of the three proteins between the Cd-treated cells and untreated cells when cells were co-treated with PD. Data represent the mean ± SEM of three independent experiments

Based on the observation of increased PCNA, H3Ser10ph, and pAurora B immunofluorescence staining, the activation of mediator proteins downstream of MAPK phosphorylation, previously found to be induced by Cd in ht-UtLM cells, was investigated in this study. We found that there were significantly increased protein expression levels of pMAPK44/42 in Cd-treated ht-UtLM cells (*P* < 0.01, Fig. 4a, b) compared to untreated control cells. As we previously reported, MAPK44/42 is highly expressed in Cd-exposed ht-UtLM cells (Gao et al. 2015; Liu et al. 2019), and this is consistent with the finding of increased expression of the proliferation maker, PCNA, in this study (see Fig. 3). We also found that increased activation of MAPK44/42 observed in Cd-treated ht-UtLM cells resulted in enhanced expression of downstream effectors pAurora B and H3Ser10ph (P < 0.01, Fig. 4A, B). Similar to PCNA expression, application of the MAPK44/42 inhibitor PD diminished expression and showed that Cd-induced increased PCNA expression correlated with MAPK44/42 activation, and subsequent downstream events of Aurora B and Histone H3 phosphorylation.

### Cd (0.1 µM and 10 µM) exposure for 10 min increased activation of FOXM1 and Cyclin D1 and their coprecipitation in ht-UtLM cells

**Fig. 5:**
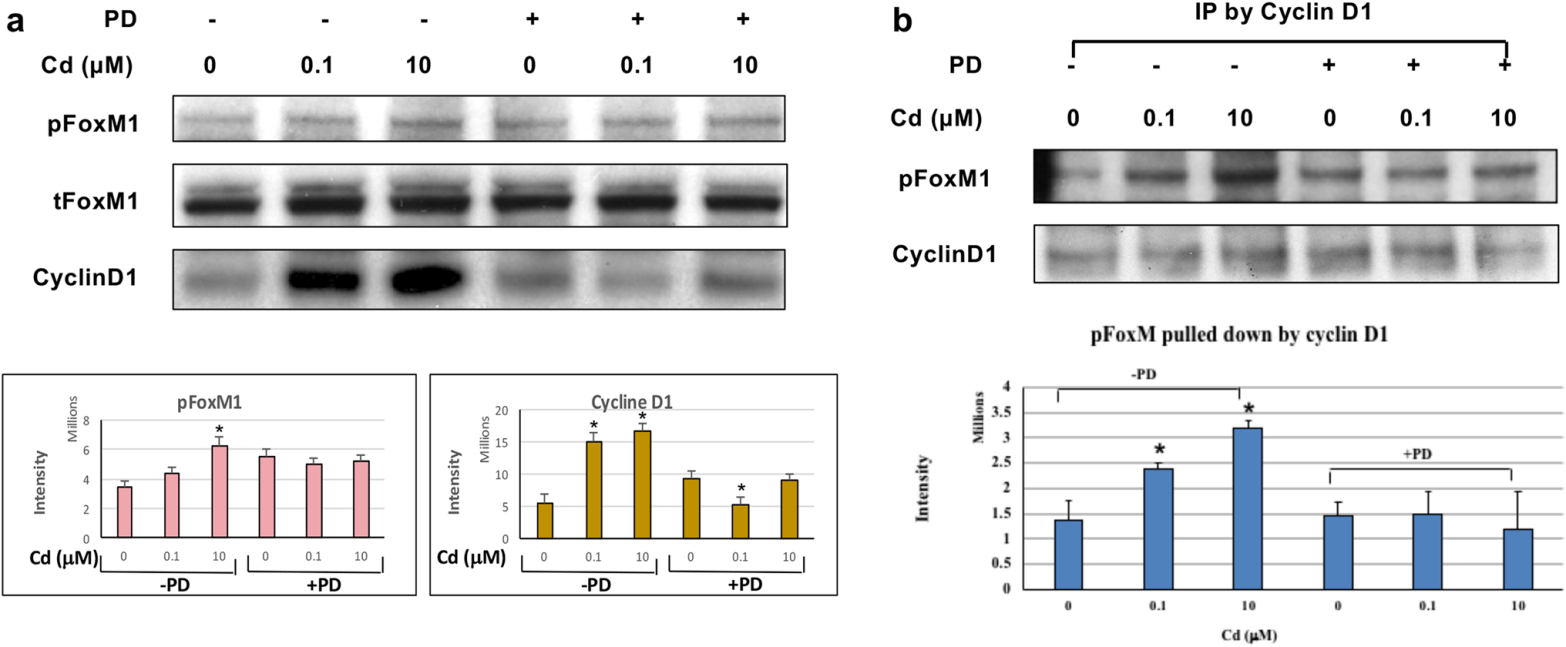
Western blots of FOXM1 and Cyclin D1, and immunoprecipitation of FOXM1 with Cyclin D1 in Cd-exposed ht-UtLM cells with or without MAPK/MEK1/2 inhibitor, PD. **a** Western blotting of FOXM1 and Cyclin D1 expression. The band intensity showed higher expression levels of pFOXM1 and Cyclin D1 in the Cd-treated ht-UtLM cells (*P < 0.01). **b** Immunoprecipitation of Cyclin D1 and pFOXM1. There is a higher level of pFOXM1 pulled down by Cyclin D1 in the Cd-treated ht-UtLM cells (*P < 0.01). PD attenuated these effects. Data represent the mean ± SEM of three independent experiments

Due to the increased expression of PCNA in Cd-exposed ht-UtLM cells, typically expressed during the S-phase of the cell cycle, and H3Ser10 expression at the metaphase plate in Cd-exposed ht-UtLM cells compared to controls, we were interested in regulators of cell cycle progression that may be regulated by MAPK to explain the differences. We used western blot analysis to determine the possible intermediary proteins downstream of MAPK that might regulate Histone H3 activation. Western blotting and immunoprecipitation were applied to determine protein expression and protein–protein interactions downstream of MAPK and upstream of Aurora B and Histone H3. MAPK regulates FOXM1, a proliferation-associated transcription factor that is involved in cell cycle regulation by cyclins, such as Cyclin D, and is immediately upstream of Aurora B. There was increased expression of phosphorylated FOXM1 and Cyclin D1 in Cd-treated cells compared to untreated cells (*p* < 0.01, Fig. 5a). In addition, increased coimmunoprecipitation of FOXM1 with Cyclin D1 was observed (*p* < 0.01, Fig. 5b) and PD diminished these effects.

### Cd (0.1 µM and 10 µM) exposure for 24 h increased colocalization of FOXM1 and Cyclin D1 in ht-UtLM cells

**Fig. 6:**
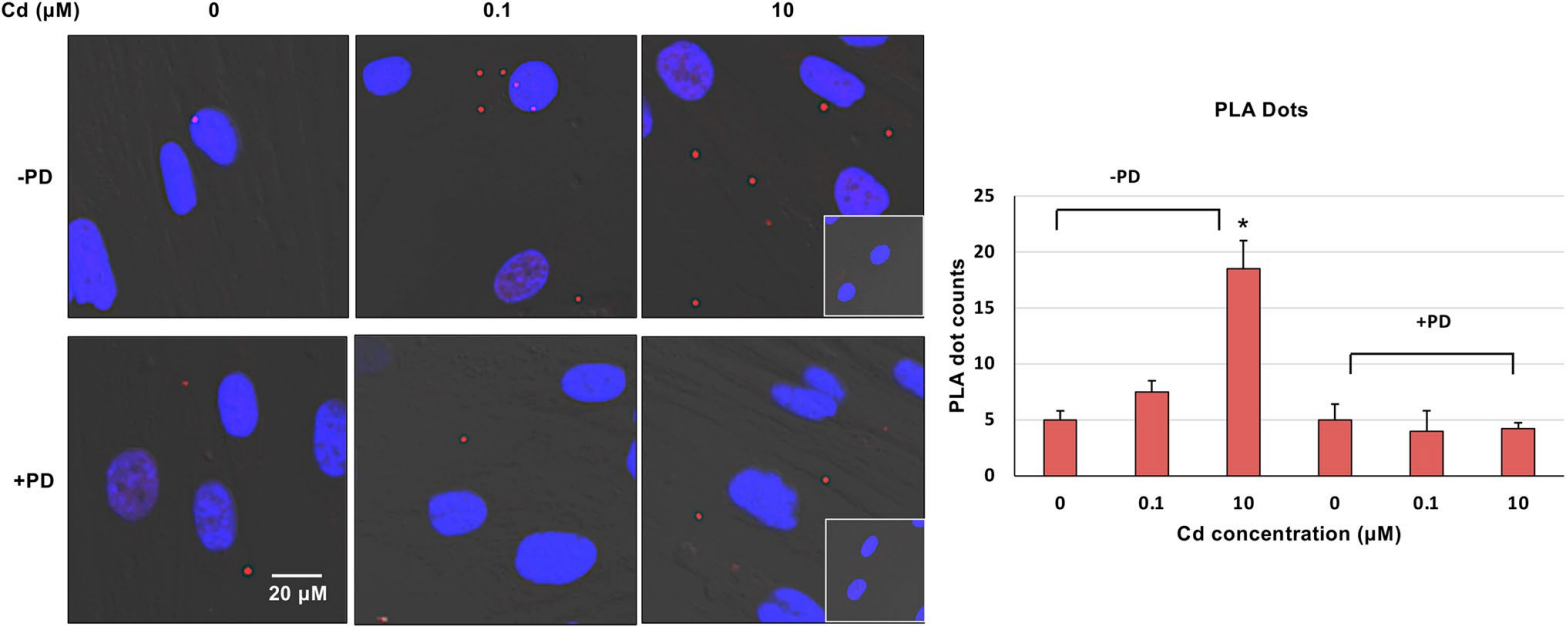
Colocalization of FOXM1 and Cyclin D1 in Cd-exposed cells **a** Proximity Ligation Assay (PLA) images. The assay detected colocalization of  FOXM1 and Cyclin D1 (red dots) under Cd treatment conditions (0.1 and 10 μM). **b** Graph of PLA dot counts. The counts of PLA dots were higher in the cells treated with Cd at 0.1 µM and significantly higher at 10 µM (*P < 0.001) compared to controls; however, the increased counts of PLA dots were diminished by MAPK/MEK1/2 inhibitor, PD administration. Data represent the mean ± SEM of three independent experiments. Inset = Negative control, only FOXM1 antibody was applied (see in “Materials and methods”)

Interaction of intermediary proteins downstream of MAPK and upstream of Aurora B was assessed following Cd exposure in ht-UtLM cells by determination of colocalization of FOXM1 and Cyclin D1 using PLA analysis. Confocal microscopy revealed the two proteins, FOXM1 and Cyclin D1 were colocalized together in the cell cytoplasm and nucleus with more PLA dots in the cells exposed to Cd compared to the controls. The increased colocalization levels were different between treatment groups of 0.1 μM (7.5) and 10 μM (18.5) compared to unexposed cells 0 μM (5.0, Fig. 6b), but only significant at 10 μM (*p* < 0.01). However, increased colocalization of FOXM1 and Cyclin D1 induced by Cd was abolished when the MAPK44/42 activation was blocked by the MAPK-specific inhibitor PD.

## Discussion

Cadmium (Cd) is a toxic element in the environment that has been associated with several disease outcomes, including cancer, even at low-dose exposures (Satarug 2018). However, the exact molecular mechanisms underlying the toxicological effects induced by low-dose Cd exposures are largely unknown and need to be elucidated. It has recently been recognized that integrative analysis of genes and proteins is required to discover biomarkers and signaling networks relevant to human toxicant exposures (Kwon et al. 2013). Therefore, the present study was designed to evaluate the molecular signaling events involved in cell proliferation, a characteristic phenomenon of tumorigenesis, induced by Cd.

Cd is described as an environmental “metalloestrogen” and its action is thought to be mediated through classical and nonclassical estrogen receptors (Johnson et al. 2003; Kluxen et al. 2012; Liu et al. 2019). Cd mimics estrogen effects in the rat uterus (Johnson et al. 2003) and Cd concentrations in blood positively correlate with ER levels and tumor volume in uteri of women with fibroids (Nasiadek et al. 2011; Ye et al. 2017). In a previous study, we found Cd stimulated growth of human uterine leiomyoma cells at lower concentrations ≤ 20 μM and this effect was not mediated by a classical ER mechanism of receptor binding and ERE-mediated gene activation, but through nongenomic pathways involving G protein-coupled estrogen receptor 1 (GPER) and activation of Epidermal Growth Factor Receptor (EGFR), with subsequent MAPK/ERK1/2 phosphorylation (Gao et al. 2015; Liu et al. 2019). In the present study, the proliferating cell nuclear antigen (PCNA), a DNA eukaryotic sliding clamp that is essential for DNA replication (Altieri and Kelman 2018), was significantly increased in Cd-exposed cells compared to unexposed Cd cells, further confirming Cd’s proliferative effects on ht-UtLM cells. The ERK1/2 (MAPK44/42) signaling cascade is one of the most extensively studied intracellular pathways and integrates a variety of extracellular stimuli into biological responses controlling cell proliferation, differentiation, or death. The cumulative evidence over the years has demonstrated the requirement of ERK in the control of cell proliferation (Chambard et al. 2007). MAPK44/42 is widely expressed and is involved in the regulation of meiosis, mitosis, and postmitotic functions in many differentiated cell types. Several different stimuli, including growth factors, cytokines, and viruses can activate the MAPK44/42 pathway (Li and Zhang 2017; Ma et al. 2010). Previously and in the present study, we have found that MAPK/MEK1/2 inhibitor, PD98059 can attenuate elevated phosphorylated MAPK44/42 expression levels in Cd-exposed ht-UtLM cells indicating that MAPK plays an important role in Cd’s proliferative effects.

Although the exact mechanism of Cd stimulation leading to uterine leiomyoma cell proliferation remains unclear, our group recently provided further evidence that Cd acting as a metal hormone could activate nongenomic estrogen receptor pathways through GPER (Liu et al. 2019). GPER and EGFR were highly expressed in human uterine leiomyoma compared to patient-matched myometrial tissues. Furthermore, in ht-UtLM cells, Cd-induced cell proliferation was inhibited by GPER-specific antagonist (G15) pretreatment, or by silencing (si) GPER, and the Cd-activated MAPK was dependent on GPER/EGFR transactivation, through significantly increased phospho-Src, matrix metalloproteinase-2 (MMP2) and MMP9, and heparin-binding EGF-like growth factor (HB-EGF) activation. All these findings indicated that ht-UtLM cell proliferation following Cd exposure was mediated through a GPR30/MMP/EGFR/MAPK signaling pathway (Liu et al. 2019). In searching for mediators downstream of MAPK44/42 activation that are important in the process of cell proliferation stimulated by Cd, we had found in earlier studies that Cd could increase the phosphorylation of Histone H3 at serine 10 in ht-UtLM cells during mitosis. Throughout mitosis in mammalian cells, chromatin condensation/decondensation is necessary for replication, repair, recombination and transcription. Histones are among the numerous DNA-binding proteins that control the level of DNA condensation. Post-translational modification of histone tails plays a critical role in the dynamic condensation/decondensation that occurs during the cell cycle. Phosphorylation of Ser10 in the tails of histone H3 has been extensively studied in many organisms, with indications that in interphase, phosphorylation of H3 correlates with chromatin (decondensation) and gene expression, whereas in mitosis it correlates with chromosome condensation. During mitosis, histone H3 is phosphorylated at Ser10 in all eukaryotes and its phosphorylation in interphase just prior to mitosis has been shown to correlate with chromosome condensation necessary for mitosis to proceed (Georgatos et al. 2009; Prigent and Dimitrov 2003). Consistently, in this study, we found there was a significant increased expression of PCNA, a marker of cell replication, and H3Ser10ph in Cd-exposed ht-UtLM cells compared to unexposed cells. However, the effects were attenuated by MAPK inhibitor PD indicating that phosphorylation of Histone H3 at serine 10 occurred downstream of MAPK activation, and H3Ser10ph was important in Cd-induced cell proliferation. It has been reported that during mitosis, H3 is phosphorylated by the kinase Aurora B and H3 phosphorylation is a hallmark of mitosis (Georgatos et al. 2009; Hirota et al. 2005). In this study, we found there was not only increased expression levels of H3Ser10ph, but also increased protein levels of phosphorylated Aurora B in Cd-exposed ht-UtLM. In addition, both Aurora B and H3Ser10ph were highly phosphorylated and localized at the metaphase plate during mitosis, which is consistent with the finding of Aurora B localization to the inner centromere, a specialized region of chromatin in early mitosis (Hindriksen et al. 2017). The effects of Cd on Aurora B phosphorylation were also blocked by MAPK inhibitor PD suggesting the phosphorylation of Aurora B occurred downstream of MAPK44/42 activation, similar to phosphorylation of Histone H3 at serine 10, when the ht-UtLM cells were exposed to Cd.

Recent studies have identified that both mitosis and autophagy are highly regulated in dynamic cellular processes involving various phosphorylation events catalyzed by kinases that play vital roles in almost all physiological and pathological conditions, such as MAPKs (mitogen-activated protein kinases), CDKs (cyclin-dependent kinases), Aurora kinases, and PI3K (phosphoinositide-3 kinase) (Li and Zhang 2017). For example, the mitogen-activated protein kinases (ERK pathway) control cell proliferation, Aurora B plays a pivotal role in cell division of the G2/M phase, and BRAF/ERK axis controls Aurora B expression at the transcriptional level, likely through the transcription factor, Fork head box M1 (FOXM1) in melanoma cells (Bonet et al. 2012). The cyclin D–CDK4/6 substrates (Cyclin D1) could contribute to the proliferative drive imposed by cyclin D–CDK4/6 activation (Di Sante et al. 2019; Major et al. 2004; Myatt and Lam 2007). Particularly, relevant example is that FOXM1 was identified as a cyclin D–CDK4/6 substrate in an unbiased proteomic screen (Anders et al. 2011; Luscher-Firzlaff et al. 2006). The multisite phosphorylation of FOXM1 by cyclin D–CDK4/6 protects FOXM1 from proteasome-mediated degradation and increases the expression of *FOXM1*-dependent target genes throughout the cell cycle to help control DNA replication, mitosis, and cell proliferation (Liao et al. 2018; Major et al. 2004; Myatt and Lam 2007). In most human cancers, FOXM1 is overexpressed, an indicator of poor prognosis for cancer patients. FOXM1 maintains cancer hallmarks by regulating the expression of target genes at the transcriptional level. Altogether, FOXM1 could act as a cyclin D–CDK4/6 substrate to initiate cell cycle progression in G2/M phase, or could act as a transcription factor to regulate target gene expression. In addition, growth factors, such as EGF that stimulate MAPK have been shown to induce cyclin D1 induction (Albanese et al. 1995). These reports prompted us to investigate the involvement of cyclin D1 and FOXM1 as a bridge downstream of MAPK and upstream of Aurora B to initiate a cascade of interactions between MAPK/FOXM1/Cyclin D/Aurora B with ultimate phosphorylation of H3 at serine 10 in Cd-induced cell proliferation of ht-UtLM cells. In this study, we did find that Cyclin D1 and FOXM1 expression was increased when ht-UtLM cells were exposed to Cd, and that the inhibition of MAPK44/42 with PD attenuated Cyclin D1 and FOXM1 expression. In addition, the association and colocalization of Cyclin D1 and FOXM1 determined by immunoprecipitation and PLA, respectively, further indicated a vital role of a Cyclin D1/FOXM1 complex in MAPK/Aurora B/H3S10ph activation induced by Cd exposure in leiomyoma cell proliferation, and presumably initiated by GPER/EGFR signaling as previously reported (Liu et al. 2019). Our results provide a molecular basis of FOXM1/cyclin D1/Aurora B signaling mediated by MAPK44/42 in cell proliferation observed following Cd exposure in ht-UtLM cells and that mediators downstream of MAPK that could initiate mitosis.

In addition to our study, there are other reports indicating that arsenic, another metal hormone, could also activate the Ras-MAPK pathway leading to independent phosphorylation of histone H3 at serine 10, and the effect could be effectively blocked by a ERK pathway inhibitor in arsenic-treated cells (Suzuki et al. 2013), a similar effect observed in our Cd study. Therefore, the phosphorylated histones H3 at serine site (H3Ser10ph) could possibly be a marker of cell proliferation induced by environmental hormonal toxicants.

## Materials and methods

### Cell culture and treatment

The ht-UtLM cells (Carney et al. 2002) were cultured in MEM (Gibco Life Technologies, Grand Island, NY) with supplements in an atmosphere of 5% CO_2_ and 95% humidity at 37 °C as previously described (Yu et al. 2008). Phenol red-free DMEM alone (PRF-DMEM; Gibco Life Technologies), along with PRF-DMEM with 10% Charcoal Dextran treated Fetal Bovine Serum (CD-FBS) (GE Healthcare Life Science Pittsburgh, PA) were used for starvation and for preparing treatment media, respectively. The cells were treated with Cd (CdCl_2_, 99%, #49800, Sigma-Aldrich, St. Louis, MO) at 0.1 and 10 μM for 10 min and 24 h for further experiments.

### MAPK/MEK1/2 inhibitor, PD98059 (PD) inhibition

A MAPK/MEK1/2 inhibitor (PD; Calbiochem, San Diego, CA) was used to detect whether the events of FOXM1/Aurora B activation leading to cell proliferation were downstream of the MAPK signaling cascade in Cd-treated cells. The treatment time and chosen concentration of PD at 30 μM was based on a titration study with the highest blocking effects and the least adverse effects on cells and PD was administered simultaneously with Cd when ht-UtLM cells were cultured for PCNA, IF, western blot, IP, and PLA studies.

### Immunofluorescence (IF) staining

IF staining was used to detect H3Ser10ph and pAurora B expression and localization in Cd-exposed ht-UtLM cells. The cells were seeded on a glass bottom 3.5 cm plate (Matsunami Glass Industry, Ltd., Osaka, Japan) at density of 30,000 cells/plate. After overnight culturing, cells were treated with Cd at 0, 0.1 or 10 µM for 24 h, and fixed with 4% paraformaldehyde for 20 min at room temperature (RT). After permeabilization with 0.5% Triton-X for 20 min at RT, the cells were incubated with a primary antibody of monoclonal clonal anti-mouse H3Ser10ph #3377, Cell Signaling Technology, Inc. Danvers, MA), and polyclonal anti-rabbit of phospho-Aurora ABC (#2914, Cell Signaling) over night at 4 °C. Negative controls received normal rabbit or mouse IgG (#ab125938 or #ab37355, Abcam, Cambridge, MA) at the same protein concentration. Then a secondary goat anti-rabbit IgG (H + L) Alexa-fluor 488 (#A-11008, Thermo Fisher Scientific, Inc. Waltham, MA) or 594 conjugated antibodies (#A-11012, Thermo Fisher Scientific, Inc.) was applied for 1 h at RT. Cells were then counterstained with 3 μM 4′,6-diamidino-2-phenylindole (DAPI) for 30 min.

IF staining was also used to detect proliferating cell nuclear antigen (PCNA) to evaluate the proliferative effects of Cd. The ht-UtLM cells were plated, cultured, and treated the same as for Aurora B staining with or without PD inhibition. The manufacturer’s protocol for PCNA staining was followed. Briefly, prior to immunostaining, the cells were fixed with 4% formaldehyde for 15 min followed by 100% methanol at -20° C for 10 min. The mouse monoclonal anti-PCNA antibody (#2586, Cell Signaling) and the goat anti-mouse IgG (H + L) Alexa-fluor 488 (#A-11008, Thermo Fisher Scientific, Inc.) as primary and secondary antibodies were applied, sequentially. The cells were then counterstained with 3 μM DAPI for 30 min.

Confocal images were taken on a Zeiss LSM710-UV (Carl Zeiss Inc, Oberkochen, Germany) using a Plan-Apochromat 63X/1.40 oil DIC M27 objective. The staining intensity was measured by Fiji, an image-processing package of ImageJ (https://fiji.sc).

### Western blot analysis

Protein extractions and western blot analyses were performed as previously described (Yu et al. 2008) to determine protein expression involved in FOXM1/Cyclin D1/Aurora B pathway activation induced by Cd. The following primary antibodies were used at 1:1000 dilution over night at 4 °C: Rabbit polyclonal anti-phospho-Aurora-ABC (#2914, Cell Signaling); Rabbit polyclonal anti-total-Aurora B (#ab2254, Abcam); Mouse monoclonal anti-phospho-Histone H3ser10 (#3377, Cell signaling); Rabbit polyclonal anti-Histone H3 (#9715, Cell Signaling); Rabbit polyclonal phospho/total FOXM1 (#14655/#5436, Cell Signaling), and Rabbit polyclonal anti-cyclin D1 (#2922, Cell Signaling). Secondary antibodies used were anti-rabbit or anti-mouse IgG Horseradish peroxidase linked (NA934V, or A931V respectively, GE Healthcare, Buckingham Shire, UK). The detection kit used was the ECL Western Blotting Detection Reagent (#RPn2106, GE Healthcare). Band intensity was obtained through a Densitometer (FluorChem™, Alpha Innotech, San-Leandro, CA).

### Immunoprecipitation (IP) studies

The IP-Kit–Dynabeads Protein G (Thermo Fisher Scientific, Waltham, MA) was used to detect the association of phospho-FOXM1 with cyclin D1. A 500 μg aliquot of total protein from cell lysate from the different conditions (see western blot analysis) was mixed with 500 μl of binding buffer. The 50 μl of Dynabeads^®^ Protein G was incubated with 10 μg of cyclin D1 rabbit polyclonal antibody (#2922, Cell Signaling) for 30 min at RT. The Dynabeads-Ab complex was then resuspended with the cell lysate and incubated on a rotation rocker at RT for 2 h. The Dynabeads-Ab-Antigen complexes were eluted according to the manufacturer’s instructions, separated on SDS-PAGE, and analyzed by western blotting at the molecular weight of FOXM1 using rabbit polyclonal anti-phospho-tyrosine (#9411, Cell Signaling) and rabbit polyclonal anti-cyclin D1 antibodies.

### Proximity ligation assays (PLA)

The PLA kit (DUO92101-1KT, Sigma-Aldrich, St. Louis, MO) was used to determine the association between FOXM1 and cyclin D1 in Cd-treated ht-UtLM cells by colocalization of these two proteins following the manufacturer’s protocol. Briefly, the cells were seeded on 3.5 cm glass bottom plate at density of 30,000 cells/plate. After ON culture, cells were treated with Cd at 0, 0.1 or 10 µM with or without PD for 24 h, fixed with 4% of paraformaldehyde for 20 min, and permeabilized with 0.1% triton X-100 for 30 min. The cells were incubated with a rabbit polyclonal anti-FOXM1 (#5436, Cell Signaling) at a 1:100 dilution and a mouse monoclonal anti-Cyclin D1 antibody (#MA1-10324, Thermo Fisher Scientific Inc.) at a 1:100 dilution, overnight at 4 °C (the cells were only incubated with FOXM1 antibody in the negative control plate). The PLUS and MINUS secondary PLA probes against rabbit and mouse IgG were added and incubated for 1 h followed by incubation with ligase for 30 min at 37 °C. Amplification was then applied for 120 min at 37 °C. The coverslips were mounted on plates with Duolink Mounting Medium with DAPI (DUO82040, Sigma-Aldrich). The cells were imaged on a Zeiss LSM780-UV (Carl Zeiss Inc, Oberkochen, Germany) using a Plan-Apochromat 40X/1.40 oil DIC M27 objective. The number of PLA dots (Bustos et al. 2017) was measured by Fiji.

### Statistical analysis

We used one-way ANOVA test with Dunnett’s post hoc comparison to compare effects (de Winter 2013) between Cd-exposed and non-exposed ht-UtLM cells in PD inhibited or uninhibited groups. We tested the difference using data from IF, IP, PLA, western band intensity and fluorescence staining intensity of PCNA. Results were represented as mean ± standard error of the mean (SEM) from three to five biological replicates. Statistical significance was defined as *p* < 0.05.

## Conclusions

**Fig. 7:**
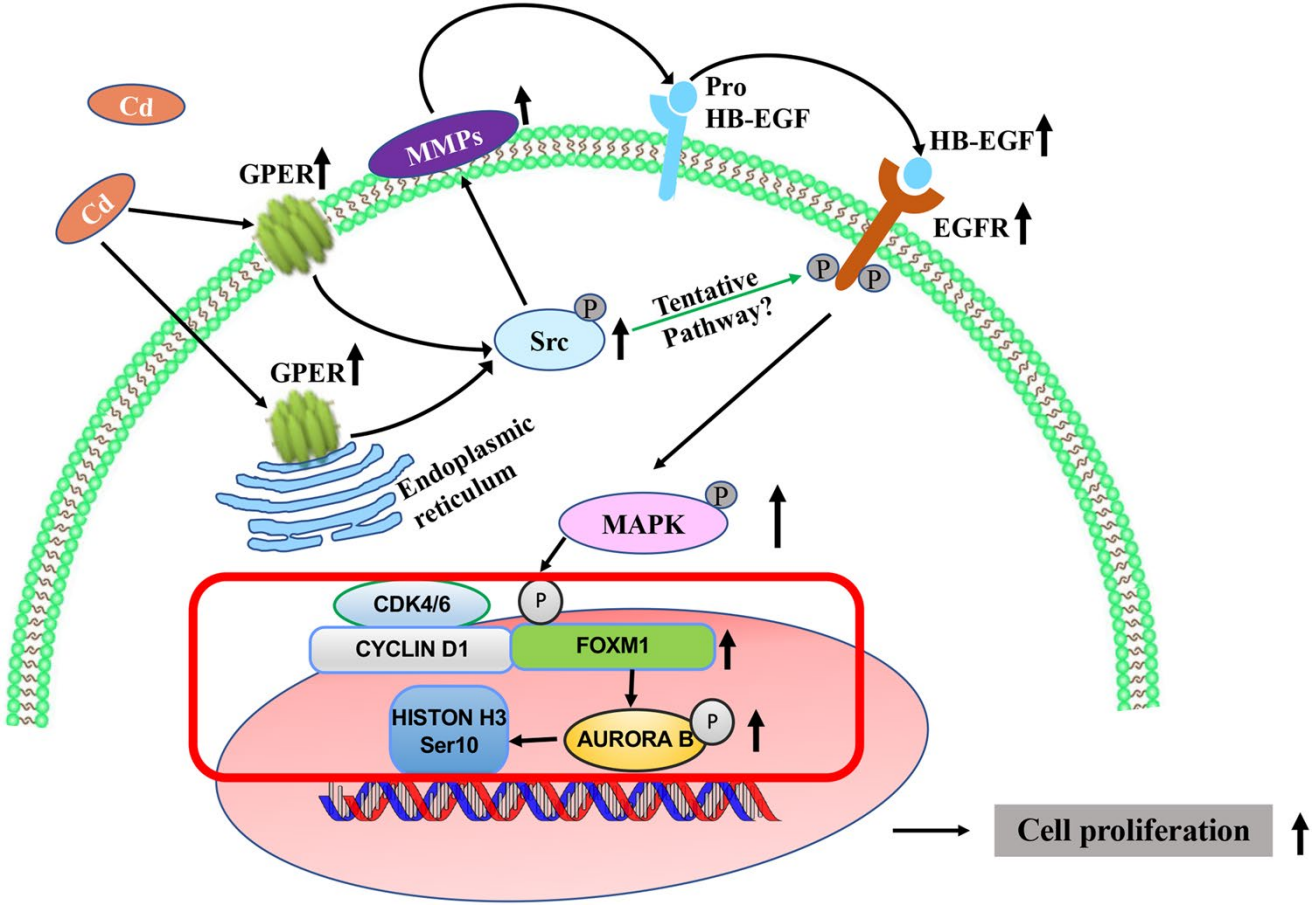
Proposed nongenomic signaling pathways involved in Cd-exposed ht-UtLM cells. Cd-induced proliferative effects observed in ht-UtLM cells have been shown to occur through the membrane/cytosolic GPER receptor via activation of the nongenomic GPER/EGFR/MAPK signaling pathway. Cd concurrently increases the expression of FOXM1 and Cyclin D1 downstream of MAPK44/42, which in turn upregulates the kinase Aurora B, leading to Histone H3 phosphorylation at the serine 10 site, and resulting in human uterine leiomyoma cell proliferation

This study provides insight into a mechanism whereby Cd, a metalloestrogen, induced cell proliferation in hormonally responsive human uterine leiomyoma (ht-UtLM) cells, and the  molecular basis of a FOXM1 and Cyclin D1-regulated event downstream of MAPK induced by Cd. The Cd-induced proliferative effects observed in ht-UtLM cells have been shown to occur through the membrane/cytosolic GPER receptor via activation of nongenomic GPER/EGFR/MAPK signaling pathway. Cd concurrently increases the expression of FOXM1 and Cyclin D1 downstream of MAPK44/42, which in turn upregulates the kinase Aurora B, leading to Histone H3 phosphorylation at serine 10 site, and resulting in human uterine leiomyoma cell mitosis (Fig. 7). Therefore, FOXM1 and Cyclin D1 appear to play pivotal roles in Cd-induced MAPK44/42/ERK1/2 cell proliferation. Human exposure to environmental concentrations of Cd, even at acceptable human exposure levels, may be an inducer of proliferation in hormonally responsive reproductive tract tumors including uterine fibroids, implicating Cd as a risk factor for humans, and particularly women with uterine fibroids.
